# Age, Resilience and Hope Predict Daily Functioning after a Flood

**DOI:** 10.1093/geroni/igaf122.3256

**Published:** 2025-12-31

**Authors:** Katie Cherry, Luke Miller, Piper Bordes, Matthew Calamia, Emily Elliott, Laura Sampson, Sandro Galea

**Affiliations:** Louisiana State University, Baton Rouge, Louisiana, United States; Louisiana State University, Baton Rouge, Louisiana, United States; Louisiana State University, Baton Rouge, Louisiana, United States; Louisiana State University, Baton Rouge, Louisiana, United States; Louisiana State University, Baton Rouge, Louisiana, United States; Stony Brook University, Stony Brook, New York, United States; Washington University in St. Louis, St. Louis, Missouri, United States

## Abstract

Prior evidence indicates that multiple flood and hurricane exposures may threaten daily functioning and psychological well-being among younger and older adults. In 2005, coastal residents from south Louisiana lost homes in Hurricane Katrina. Many permanently relocated to the greater Baton Rouge region, an inland community on presumably higher and safer ground. In August of 2016, historic flooding brought widespread destruction across a 22-parish (county) area, creating a second round of catastrophic losses for those who had moved to Baton Rouge after Katrina. The present research is part of a larger study on health and well-being after multiple disaster exposures. Here we examined associations among self-reported daily functioning indexed by the Barkley Functional Impairment Scale (BFIS) and resilience and risk factors within the first two years of the flood. Three flood exposure groups were compared: non-flooded (controls), single disaster (flooded in 2016) and double disaster (flooded in 2005 and again in 2016). Results indicated that the flood exposure groups did not differ in self-reported daily functioning, although symptoms of depression and post-traumatic stress were greater for those whose homes flooded relative to the non-flooded controls. Regression analyses indicted that age, resilience, hope, depression, and PTS symptoms predicted daily functioning after key sociodemographic factors, flood stressors, and prior lifetime trauma were accounted for. Strategies for managing the long-term impacts of multiple disasters for people who live in geographic areas that are prone to severe weather events are discussed.

